# The importance of negative determinants as modulators of CK2 targeting. The lesson of Akt2 S131

**DOI:** 10.1371/journal.pone.0193479

**Published:** 2018-03-01

**Authors:** Jordi Vilardell, Cristina Girardi, Oriano Marin, Giorgio Cozza, Lorenzo A. Pinna, Maria Ruzzene

**Affiliations:** 1 Department of Biomedical Sciences, University of Padova, Padova, Italy; 2 Department of Molecular Medicine, University of Padova, Padova, Italy; 3 CNR Neuroscience Institute, Padova, Italy; Universite du Quebec a Trois-Rivieres, CANADA

## Abstract

CK2 is a pleiotropic S/T protein kinase (formerly known as casein kinase 2) which is attracting increasing interest as therapeutic target, and the identification of its substrates is a crucial step in determining its involvement in different pathological conditions. We recently found that S131 of Akt2 (homologous to the well established CK2 target S129 of Akt1) is not phosphorylated by CK2 either in vitro or in vivo, although the consensus sequence recognized by CK2 (S/T-x-x-E/D/pS/pT) is conserved in it. Here, by exploiting synthetic peptides, in cell transfection experiments, and computational analysis, we show that a single sequence element, a T at position n+1, hampers phosphorylation, causing an α-helix structure organization which prevents the recognition of its own consensus by CK2. Our results highlight the role of negative determinants as crucial modulators of CK2 targeting and corroborate the concept that Akt1 and Akt2 display isoform specific features. Experiments with synthetic peptides suggest that Akt2 S131 could be phosphorylated by kinases of the Plk (Polo-like kinase) family, which are insensitive to the presence of the n+1 T. The low phylogenetic conservation of the Akt2 sequence around S131, as opposed to the extremely well-conserved Akt1 homologous sequence, would indicate a dominant positive role in the selective pressure only for the Akt1 phosphoacceptor site committed to undergo phosphorylation by CK2. By contrast, Akt2 S131 may mediate the response to specific physio/pathological conditions, being consequently shielded against basal CK2 targeting.

## Introduction

CK2 is an acidophylic S/T protein kinase that phosphorylates hundreds of proteins involved in almost all cellular processes [1]. The phosphorylation of many substrates mediate a global anti-apoptotic function of CK2 [2], and in fact its overexpression is observed in pathological conditions characterized by abnormal cell survival, cancer in primis. Moreover, there are CK2 substrates related to neurodegenerative disorders [3–5] and other pathologies [6–10], and the enzyme is attracting increasing interest as a promising therapeutic target.

The minimum consensus sequence recognized by CK2 (S/T-x-x-E/D/pX) has been known for decades [11]. To note that the crucial acidic side chain at position n+3 can be not only that of a carboxylic aminoacid (Glu or Asp) but also a phospho-residue (pX) generated by another kinase or by CK2 itself, a feature this latter referred to as “hierarchical phosphorylation” [12]. Beside the minimal requirement, numerous other elements have been identified as important in determining phosphorylation by CK2; in general, additional acidic side chains act as positive determinants, especially downstream at positions up to n+7 (or even farther) relative to the target residue. On the contrary, basic residues are detrimental. Moreover, a Pro in n+1 (which is an essential requirement for Pro-directed kinases) acts as a negative determinant for CK2, hampering the phosphorylation of even very acidic sequences [13]. Most of this information has been collected from studies with synthetic peptides [14–17], integrated by mutational analysis of CK2 critical sites [18,19]. More recently, the CK2 consensus has been definitely confirmed by the continuously growing number of phospho-sites identified as CK2 substrates *in vitro* and *in vivo*. Their sequences, retrieved in databases such as Phosphosite-plus (http://www.phosphosite.org/) or phosphoElm (http://phospho.elm.eu.org/), allowed the generation of weblogos [20] where acidic residues are largely predominant at all positions with special reference to n+3 and n+1, basic residues are nearly absent and the same applies to a Pro at position n+1.

On all these bases, the identification of CK2 sites from protein sequence is usually considered quite predictable and very reliable, provided that the region is exposed to the protein surface. Akt (also known as PKB) is one of the crucial CK2 substrates: a part from an intricate network of connections between the two kinase pathways [21], CK2 directly phosphorylates Akt [22], and this boosts its functions, mainly related to survival, migration and proliferation. The potentiation of Akt signaling by CK2 phosphorylation is due not only to a direct effect, but also to a protection against the dephosphorylation of the up-regulatory site pT308 [23].

The two main ubiquitous isoforms of Akt are Akt1 (or PKBα) and Akt2 (or PKBβ). The CK2 phosphorylation site on Akt1 fulfills all the canonical rules. It is represented by S129, in the linker region of Akt1, between the PH and the catalytic domains, and it is readily phosphorylated by CK2 both *in vitro* and *in vivo* [22,23]. Indeed, direct phosphorylation of Akt1 at S129 is widely exploited as a reporter of endogenous CK2 activity in living cells [24] and commercial phosphor-specific antibodies against this phospho-residue are available for this purpose.

In Akt2, the residue homologous to Akt1 S129 is S131. It is also a perfect CK2 consensus site (see below, Tables 1, 2 and 3), but, surprisingly, we have recently demonstrated that it is refractory to phosphorylation by CK2 [25] and, accordingly, Akt2 S131 phosphorylation has never been reported in phosphosite databases (the only reported case on PhosphoSite-plus has been mis-inferred from our paper [25], where we observed a weak in vitro phosphorylation only upon thermal denaturation of Akt2 protein).

**Table 1:** Comparison of Akt1 sequences in different species. Analysis was performed by means of Basic Local Alignment Search Tool (BLAST) software of Uniprot. A 29-aminoacid fragment around S129 Akt1 of human sequences was used as reference. Invariable residues at positions between n-3 and n+3 are in bold, while variations are underlined.

AKT1 (residues 110-146)																	129															* *
																	|															* *
			E	E	M	D	F	R	S	G	-	S	P	S	D	N	S	G	A	E	E	M	E	V	S	L	A	K	P	K	H	* *
Uniprot Ref.	Scientific name	Common name																														* *
P31749	*Homo sapiens*	Human	E	E	M	D	F	R	S	G	-	S	P	**S**	**D**	**N**	**S**	**G**	**A**	**E**	E	M	E	V	S	L	A	K	P	K	H	*143*
K7C9N2	*Pan troglodytes*	Chimpanzee	E	E	M	D	F	R	S	G	-	S	P	**S**	**D**	**N**	**S**	**G**	**A**	**E**	E	M	E	V	S	L	A	K	P	K	H	*143*
G1S2V0	*Nomascus leucogenys*	Northern white-cheeked gibbon	E	E	M	D	F	R	S	G	-	S	P	**S**	**D**	**N**	**S**	**G**	**A**	**E**	E	M	E	V	S	L	A	K	P	K	H	*143*
G3RB32	*Gorilla gorilla gorilla*	Western lowland gorilla	E	E	M	D	F	R	S	G	-	S	P	**S**	**D**	**N**	**S**	**G**	**A**	**E**	E	M	E	V	S	L	A	K	P	K	H	*143*
A0A1U7TIP6	*Tarsius syrichta*	Philippine tarsier	E	M	M	D	F	R	S	G	-	S	P	**S**	**D**	**N**	**S**	**G**	**A**	**E**	E	M	E	V	S	L	A	K	P	K	H	*143*
P31750	*Mus musculus*	Mouse	E	T	M	D	F	R	S	G	-	S	P	**S**	**D**	**N**	**S**	**G**	**A**	**E**	E	M	E	V	S	L	A	K	P	K	H	*143*
A0A1U7QIL2	*Mesocricetus auratus*	Golden hamster	E	T	M	D	F	R	S	G	-	S	P	**S**	**D**	**N**	**S**	**G**	**A**	**E**	E	M	E	V	S	L	A	K	P	K	H	*143*
F7HW94	*Callithrix jacchus*	White-tufted-ear marmoset	E	M	M	D	F	R	S	G	-	S	P	**S**	**D**	**N**	**S**	**G**	**A**	**E**	E	M	E	V	S	L	A	K	P	R	H	*141*
I3ML41	*Ictidomys tridecemlineatus*	Thirteen-lined ground squirrel	E	M	M	D	F	R	S	G	-	S	P	**S**	**D**	**N**	**S**	**G**	**A**	**E**	E	M	E	V	S	L	A	K	P	K	H	*143*
M3XST6	*Mustela putorius furo*	European domestic ferret	E	M	M	D	F	R	S	G	-	S	P	**S**	**D**	**N**	**S**	**G**	**A**	**E**	E	M	E	V	S	L	A	K	P	K	H	*143*
A0A143RHU3	*Felis catus*	Cat	E	M	M	D	F	R	S	G	-	S	P	**S**	**D**	**N**	**S**	**G**	**A**	**E**	E	M	E	V	S	L	A	K	P	K	H	*143*
G1LMQ4	*Ailuropoda melanoleuca*	Giant panda	E	M	M	D	F	R	S	G	-	S	P	**S**	**D**	**N**	**S**	**G**	**A**	**E**	E	M	E	V	S	L	A	K	P	K	H	*143*
E2RJX4	*Canis lupus familiaris*	Dog	E	M	M	D	F	R	S	G	-	S	P	**S**	**D**	**N**	**S**	**G**	**A**	**E**	E	M	E	V	S	L	A	K	P	K	H	*143*
U3C443	*Callithrix jacchus*	White-tufted-ear marmoset	E	M	M	D	F	R	S	G	-	S	P	**S**	**D**	**N**	**S**	**G**	**A**	**E**	E	M	E	V	S	L	A	K	P	R	H	*143*
F6SAS5	*Macaca mulatta*	Rhesus macaque	E	M	M	D	F	R	S	G	-	S	P	**S**	**D**	**N**	**S**	**G**	**A**	**E**	E	M	E	V	S	L	A	K	P	K	H	*143*
A0A096NK32	*Papio anubis*	Olive baboon	E	M	M	D	F	R	S	G	-	S	P	**S**	**D**	**N**	**S**	**G**	**A**	**E**	E	M	E	V	S	L	A	K	P	K	H	*143*
A0A0D9RBG0	*Chlorocebus sabaeus*	Green monkey	E	M	M	D	F	R	S	G	-	S	P	**S**	**D**	**N**	**S**	**G**	**A**	**E**	E	M	E	V	S	L	A	K	P	K	H	*143*
F6WGH2	*Equus caballus*	Horse	E	M	-	D	F	R	S	G	-	S	P	**S**	**D**	**N**	**S**	**G**	**A**	**E**	E	M	E	V	S	L	A	K	P	K	H	*142*
P47196	*Rattus norvegicus*	Rat	E	T	M	D	F	R	S	G	-	S	P	**S**	**D**	**N**	**S**	**G**	**A**	**E**	E	M	E	V	A	L	A	K	P	K	H	*143*
C1PIG3	*Sus scrofa*	Pig	E	M	M	D	F	R	S	G	-	S	P	**S**	**E**	**N**	**S**	**G**	**A**	**E**	E	M	E	V	S	L	A	K	P	K	H	*143*
H0XPQ9	*Otolemur garnettii*	Small-eared galago	E	M	M	D	F	R	S	G	-	S	P	**S**	**D**	**N**	**S**	**G**	**A**	**E**	E	M	E	V	A	L	A	K	P	R	H	*143*
A0A1S2ZF54	*Erinaceus europaeus*	Western European hedgehog	E	M	M	D	F	H	S	G	-	S	P	**S**	**D**	**N**	**S**	**G**	**A**	**E**	E	M	E	V	A	L	A	K	P	K	H	*144*
Q01314	*Bos taurus*	Bovine	E	T	M	D	F	R	S	G	-	S	P	**G**	**E**	**N**	**S**	**G**	**A**	**E**	E	M	E	V	S	L	A	K	P	K	H	*143*
D9IA52	*Capra hircus*	Goat	E	T	M	D	F	R	S	G	-	S	P	**G**	**E**	**N**	**S**	**G**	**A**	**E**	E	M	E	V	S	L	A	K	P	K	H	*143*
T1WF18	*Anser anser*	domestic goose	E	M	M	D	F	R	S	G	-	S	P	**S**	**D**	**N**	**S**	**G**	**A**	**E**	E	M	E	V	S	M	T	K	P	K	H	*36*
A0A1U7S7Y2	*Alligator sinensis*	Chinese alligator	E	M	M	D	F	R	S	G	-	S	P	**S**	**D**	**N**	**S**	**G**	**A**	**E**	E	M	E	V	S	M	T	K	P	K	H	*81*
G1NL49	*Meleagris gallopavo*	Common turkey	E	M	M	D	F	R	S	G	-	S	P	**S**	**D**	**N**	**S**	**G**	**A**	**E**	E	M	E	V	S	M	T	K	P	K	H	*143*
K7FKB4	*Pelodiscus sinensis*	Chinese softshell turtle	E	M	M	D	F	R	S	G	-	S	P	**S**	**D**	**N**	**S**	**G**	**A**	**E**	E	M	E	V	S	M	T	K	P	K	H	*143*
F1NMS0	*Gallus gallus*	Chicken	E	M	M	D	F	R	S	G	-	S	P	**S**	**D**	**N**	**S**	**G**	**A**	**E**	E	M	E	V	S	M	T	K	P	K	H	*143*
U3JJZ0	*Ficedula albicollis*	Collared flycatcher	E	M	M	D	F	R	S	G	-	S	P	**S**	**D**	**N**	**S**	**G**	**A**	**E**	E	M	E	V	S	M	T	K	P	K	H	*143*
U3J9Q1	*Anas platyrhynchos*	Mallard	E	M	M	D	F	R	S	G	-	S	P	**S**	**D**	**N**	**S**	**G**	**A**	**E**	E	M	E	V	S	M	T	K	P	K	H	*144*
H0ZRP6	*Taeniopygia guttata*	Zebra finch	E	M	M	D	F	R	S	G	-	S	P	**S**	**D**	**N**	**S**	**G**	**A**	**E**	E	M	E	V	S	M	T	K	P	K	H	*147*
H0V394	*Cavia porcellus*	Guinea pig	E	L	M	E	F	Q	S	G	-	S	P	**S**	**D**	**N**	**S**	**G**	**A**	**E**	E	M	E	V	S	L	A	K	P	K	H	*143*
A0A0P6JFJ2	*Heterocephalus glaber*	Naked mole rat	E	L	M	E	F	Q	S	G	-	S	P	**S**	**D**	**N**	**S**	**G**	**A**	**E**	E	M	E	V	S	L	A	K	P	K	H	*143*
G3W4L9	*Sarcophilus harrisii*	Tasmanian devil	E	L	M	D	F	R	S	G	-	S	P	**S**	**D**	**N**	**S**	**G**	**A**	**E**	E	M	E	V	S	T	T	K	P	K	H	*143*
A0A1S3EV04	*Dipodomys ordii*	Ord's kangaroo rat	-	T	M	D	F	R	S	G	-	S	P	**S**	**D**	**N**	**S**	**G**	**A**	**E**	E	M	E	V	S	L	A	K	P	K	H	*142*
G1KLB6	*Anolis carolinensis*	Green anole	E	I	M	D	F	R	S	G	-	S	P	**S**	**D**	**N**	**S**	**G**	**A**	**E**	E	M	E	I	S	M	T	K	P	K	H	*143*
F6QP11	*Monodelphis domestica*	Gray short-tailed opossum	E	L	M	D	F	Q	S	G	-	S	P	**S**	**D**	**N**	**S**	**G**	**A**	**E**	E	M	E	V	S	T	T	K	P	K	H	*143*
Q98TY9	*Xenopus laevis*	African clawed frog	E	M	M	E	V	R	S	G	D	S	P	**S**	**D**	**N**	**S**	**G**	**A**	**E**	E	M	E	V	S	H	S	K	P	K	H	*144*
F6XP81	*Xenopus tropicalis*	Western clawed frog	E	M	M	E	V	R	S	G	D	S	P	**S**	**D**	**N**	**S**	**G**	**A**	**E**	E	M	E	V	S	H	S	K	P	K	H	*148*
H3A5T1	*Latimeria chalumnae*	West Indian ocean coelacanth	E	M	M	D	F	N	S	-	-	-	P	**S**	**D**	**N**	**S**	**D**	**A**	**G**	E	M	E	V	S	L	T	R	P	K	H	*141*
W5N501	*Lepisosteus oculatus*	Spotted gar	E	M	M	D	C	R	S	G	-	S	P	**S**	**D**	**N**	**I**	**G**	**I**	**E**	D	M	E	V	Y	L	T	K	P	R	L	*143*

**Table 2:** Comparison of Akt2 sequences in different species. Analysis was performed by means of Basic Local Alignment Search Tool (BLAST) software of Uniprot. A 29-aminoacid fragment around S131 Akt2 of human sequences was used as reference. Invariable residues at positions between n-3 and n+3 are in bold, while variations are underlined. The common name of species with T at n+1 position, as in human, are underlined.

AKT2 (residues 117-145)																	131															
																	|															
			E	D	P	M	D	Y	K	C	G	S	P	S	D	S	S	T	T	E	E	M	E	V	A	V	S	K	A	R	A	* *
Uniprot Ref.	Scientific name	Common name																														
P31751	*Homo sapiens*	*Human*	E	D	P	M	D	Y	K	C	G	S	P	**S**	**D**	**S**	**S**	**T**	**T**	**E**	E	M	E	V	A	V	S	K	A	R	A	*145*
K7CDC2	*Pan troglodytes*	*Chimpanzee*	E	D	P	M	D	Y	K	C	G	S	P	**S**	**D**	**S**	**S**	**T**	**A**	**E**	E	M	E	V	A	V	S	K	A	R	A	*145*
H2NYU6	*Pongo abelii*	*Sumatran orangutan*	E	D	P	M	D	Y	K	C	G	S	P	**S**	**D**	**S**	**S**	**T**	**A**	**E**	E	M	E	V	A	V	S	K	A	R	A	*145*
G3QGC8	*Gorilla gorilla gorilla*	*Western lowland gorilla*	E	D	P	M	D	Y	K	C	G	S	P	**S**	**D**	**S**	**S**	**T**	**A**	**E**	E	M	E	V	A	V	S	K	A	R	A	*145*
H0VK35	*Cavia porcellus*	*Guinea pig*	E	D	P	M	D	Y	K	C	G	S	P	**S**	**D**	**S**	**S**	**T**	**T**	**E**	E	M	E	V	A	V	S	K	A	R	A	*145*
A0A1D5RH80	*Macaca mulatta*	*Rhesus macaque*	E	D	P	M	D	Y	K	C	G	S	P	**S**	**D**	**S**	**S**	**A**	**A**	**E**	E	M	E	V	A	V	S	K	A	R	A	*83*
F6XQD8	*Macaca mulatta*	*Rhesus macaque*	E	D	P	M	D	Y	K	C	G	S	P	**S**	**D**	**S**	**S**	**A**	**A**	**E**	E	M	E	V	A	V	S	K	A	R	A	*145*
I3M1D1	*Ictidomys tridecemlineatus*	*Thirteen-lined ground squirrel*	E	D	P	M	D	Y	K	C	G	S	P	**S**	**D**	**S**	**S**	**T**	**A**	**E**	E	M	E	V	A	V	S	K	A	R	A	*130*
A0A0D9QUT3	*Chlorocebus sabaeus*	*Green monkey*	E	D	P	M	D	Y	K	C	G	S	P	**S**	**D**	**S**	**S**	**A**	**A**	**E**	E	M	E	V	A	V	S	K	A	R	A	*145*
G1SD70	*Oryctolagus cuniculus*	*Rabbit*	E	D	P	M	D	Y	K	C	G	S	P	**S**	**D**	**S**	**S**	**A**	**A**	**E**	E	M	E	V	A	V	S	K	A	R	A	*145*
S9YPT4	*Camelus ferus*	*Wild bactrian camel*	E	D	P	M	D	Y	K	C	G	S	P	**S**	**D**	**S**	**S**	**A**	**A**	**E**	E	M	E	V	A	V	S	K	A	R	A	*89*
M9WQH0	*Bubalus bubalis*	*Domestic water buffalo*	E	D	P	M	D	Y	K	C	G	S	P	**S**	**D**	**S**	**S**	**A**	**A**	**E**	E	M	E	V	A	V	S	K	A	R	A	*145*
G3SRC6	*Loxodonta africana*	*African elephant*	E	D	P	M	D	F	K	C	G	S	P	**S**	**D**	**S**	**S**	**T**	**A**	**E**	E	M	E	V	A	V	S	K	A	R	A	*145*
F6RGP1	*Equus caballus*	*Horse*	E	D	P	M	D	Y	K	C	G	S	P	**S**	**D**	**S**	**S**	**A**	**A**	**E**	E	M	E	V	A	V	S	K	A	R	A	*145*
0A1U7U3E7	*Tarsius syrichta*	*Philippine tarsier*	E	D	P	M	D	Y	K	C	G	S	P	**S**	**D**	**S**	**S**	**A**	**A**	**E**	E	M	E	V	A	V	S	K	A	R	A	*150*
H0XA83	*Otolemur garnettii*	*Small-eared galago*	K	D	P	M	D	Y	K	C	G	S	P	**S**	**D**	**S**	**S**	**T**	**A**	**E**	E	M	E	V	A	V	S	K	A	R	A	*145*
F7HW88	*Callithrix jacchus*	*White-tufted-ear marmoset*	E	D	P	M	D	Y	K	C	G	S	P	**S**	**D**	**S**	**S**	**V**	**A**	**E**	E	M	E	V	A	V	S	K	A	R	A	*145*
F7I4A2	*Callithrix jacchus*	*White-tufted-ear marmoset*	E	D	P	M	D	Y	K	C	G	S	P	**S**	**D**	**S**	**S**	**V**	**A**	**E**	E	M	E	V	A	V	S	K	A	R	A	*145*
P47197	*Rattus norvegicus*	*Rat*	E	D	A	M	D	Y	K	C	G	S	P	**S**	**D**	**S**	**S**	**T**	**S**	**E**	M	M	E	V	A	V	S	K	A	R	A	*145*
E1B9D1	*Bos taurus*	*Bovine*	D	D	P	M	D	Y	K	C	G	S	P	**S**	**D**	**S**	**S**	**A**	**A**	**E**	E	M	E	V	A	V	S	K	A	R	A	*145*
F1PRG4	*Canis lupus familiaris*	*Dog*	E	D	P	M	D	Y	K	C	G	S	P	**S**	**D**	**P**	**S**	**A**	**A**	**E**	E	M	E	V	A	V	S	K	A	R	A	*145*
M3Z1Y7	*Mustela putorius furo*	*European domestic ferret*	E	D	P	M	D	Y	K	C	G	S	P	**S**	**D**	**P**	**S**	**A**	**A**	**E**	E	M	E	V	A	V	S	K	A	R	A	*145*
M3WGH1	*Felis catus*	*Cat*	E	D	P	M	D	Y	K	C	G	S	P	**S**	**D**	**P**	**S**	**A**	**A**	**E**	E	M	E	V	A	V	S	K	A	R	A	*145*
G1M1Q8	*Ailuropoda melanoleuca*	*Giant panda*	E	D	P	M	D	Y	K	C	G	S	P	**S**	**D**	**P**	**S**	**A**	**A**	**E**	E	M	E	V	A	V	S	K	A	R	A	*145*
F1PSA7	*Canis lupus familiaris*	*Dog*	E	D	P	M	D	Y	K	C	G	S	P	**S**	**D**	**P**	**S**	**A**	**A**	**E**	E	M	E	V	A	V	S	K	A	R	A	*145*
A0A0B8RZS0	*Sus scrofa domesticus*	*domestic pig*	E	D	P	M	D	Y	K	C	G	S	P	**S**	**D**	**S**	**S**	**A**	**A**	**E**	E	M	E	V	A	V	S	K	A	R	A	*145*
Q60823	*Mus musculus*	*Mouse*	E	D	A	M	D	Y	K	C	G	S	P	**S**	**D**	**S**	**S**	**T**	**S**	**E**	M	M	E	V	A	V	N	K	A	R	A	*145*
U6D7I2	*Neovison vison*	*Mustela vison*	E	D	P	M	D	F	K	C	G	S	P	**S**	**D**	**P**	**S**	**A**	**A**	**E**	E	M	E	V	A	V	S	K	A	R	A	*69*
A0A1S3GIW6	*Dipodomys ordii*	*Ord's kangaroo rat*	E	D	S	M	D	Y	K	C	G	S	P	**S**	**D**	**S**	**S**	**T**	**P**	**E**	E	M	D	V	A	V	S	R	A	R	A	*145*
H2QGC2	*Pan troglodytes*	*Chimpanzee*	E	D	P	M	D	Y	K	C	G	S	P	**S**	**D**	**S**	**S**	**T**	**A**	**E**	E	M	E	V	A	V	A	K	-	H	G	*48*
G1NXG4	*Myotis lucifugus*	*Little brown bat*	E	D	P	M	E	Y	K	C	G	S	P	**G**	**D**	**S**	**S**	**A**	**A**	**E**	E	M	E	V	A	V	S	K	A	R	A	*145*
F7BKI3	*Monodelphis domestica*	*Gray short-tailed opossum*	E	D	L	M	D	Y	K	C	G	S	P	**S**	**D**	**S**	**S**	**V**	**A**	**E**	D	M	E	V	A	V	S	K	T	R	T	*145*
H9G6X3	*Anolis carolinensis*	*Green anole*	E	D	R	M	D	Y	K	C	G	S	P	**S**	**D**	**S**	**T**	**G**	**A**	**E**	E	M	E	V	A	V	T	K	T	R	A	*145*
A0A1S3ALD3	*Erinaceus europaeus*	*Western European hedgehog*	K	A	-	-	-	Y	E	C	G	S	P	**S**	**D**	**S**	**S**	**A**	**A**	**E**	E	M	E	V	A	V	S	K	A	R	A	*145*
A0A1U8CNN2	*Mesocricetus auratus*	*Golden hamster*	D	S	A	I	D	Y	K	C	G	S	P	**S**	**D**	**S**	**S**	**P**	**S**	**E**	M	M	E	V	A	V	N	K	A	R	A	*142*
A0A1U7SGJ4	*Alligator sinensis*	*Chinese alligator*	E	D	A	M	D	Y	K	C	G	S	P	**N**	**D**	**S**	**A**	**G**	**A**	**E**	E	M	E	V	A	V	T	K	T	R	V	*102*
W5NBQ5	*Lepisosteus oculatus*	*Spotted gar*	E	E	P	M	D	I	K	F	G	S	P	**S**	**D**	**N**	**S**	**G**	**A**	**E**	E	M	E	V	A	M	S	K	S	R	S	*145*
A0A087XMG3	*Poecilia formosa*	*Amazon molly*	E	E	P	M	E	I	K	F	G	S	P	**S**	**D**	**S**	**S**	**G**	**A**	**E**	E	M	E	I	A	M	C	K	S	R	S	*145*
M4ADD6	*Xiphophorus maculatus*	*Southern platyfish*	E	E	P	M	E	I	K	F	G	S	P	**S**	**D**	**S**	**S**	**G**	**A**	**E**	E	M	E	I	A	M	C	K	S	R	S	*146*
H3BHT2	*Latimeria chalumnae*	*West Indian ocean coelacanth*	E	S	-	M	D	F	K	C	G	S	P	**N**	**D**	**S**	**M**	**G**	**A**	**E**	E	M	E	V	V	V	S	K	T	H	V	*145*
F6W6V1	*Ornithorhynchus anatinus*	*Duckbill platypus*	E	D	S	M	E	F	K	C	S	S	P	**G**	**D**	**P**	**S**	**A**	**A**	**E**	E	M	E	V	A	V	T	K	T	R	T	*147*
Q7ZX15	*Xenopus laevis*	*African clawed frog*	E	E	A	M	E	V	K	Y	G	S	P	**S**	**D**	**V**	**S**	**S**	**A**	**E**	Q	M	D	V	A	M	S	K	G	H	P	*150*
B1WB72	*Xenopus tropicalis*	*Western clawed frog*	E	E	A	M	E	V	K	Y	G	S	P	**S**	**D**	**V**	**S**	**S**	**A**	**E**	Q	M	D	V	A	M	S	K	G	R	P	*149*
Q6IP76	*Xenopus laevis*	*African clawed frog*	E	E	A	M	E	V	K	Y	G	S	P	**S**	**D**	**V**	**S**	**S**	**A**	**E**	Q	M	D	V	A	M	S	K	G	R	P	*149*

**Table 3:** List of the peptides used in this work, with sequence (one-letter code) and nomenclature adopted. The parental peptides are indicated as wild type (wt). The CK2 target site Akt1 S129 and its homologous residue Akt2 S131 are underlined. Positions not conserved in the two Akt isoforms (human sequence), and substitutions that swap Akt1/Akt2 positions in the variant peptides, are in bold. The same colors denote substitutions that swap Akt1/Akt2 positions. Green indicates other substitutions. Three N-terminal Arginine residues (R3) were introduced for technical reasons (see Methods).

Peptide name	Sequence
**Akt1**(wt)	R_3_GSPSD**N**S**GA**EEMEV
**Akt2**(wt)	R_3_GSPSD**S**S**TT**EEMEV
**Akt2N**	R_3_GSPSD**N**S**TT**EEMEV
**Akt2A**	R_3_GSPSD**S**S**TA**EEMEV
**Akt2TG**	R_3_GSPSD**S**S**GT**EEMEV
**Akt2TA**	R_3_GSPSD**S**S**AT**EEMEV
**Akt2TS**	R_3_GSPSD**S**S**ST**EEMEV
**Akt1GT**	R_3_GSPSD**N**S**TA**EEMEV

One-letter code for the aminoacids of the above peptides: A = Ala = alanine; D = Asp = aspartic acid; E = Glu = glutamic acid; G = Gly = glycine; M = Met = methionine; N = Asn = asparagine; P = Pro = proline; R = Arg = arginine; S = Ser = serine; T = Thr = threonine; V = Val = valine

To shed light on the molecular features underlying this apparent paradox, we performed a study aimed at understanding whether the different susceptibility of Akt1 and Akt2 to phosphorylation by CK2 depends on sequence determinants or on higher structural elements. The results are described in this report.

## Materials and methods

### Enzymes

Recombinant CK2α and CK2 α_2_β_2_ were expressed, purified and kindly donated by Dr. Stefania Sarno (Padova, Italy) and Andrea Venerando (Padova, Italy).

Recombinant human PLK3-GST (Polo-like kinase3 - Glutathione S-transferase), cloned in PGEX-4T1 vector, was produced in BL21 cells and purified by glutathione-Sepharose affinity chromatography, eluted by an elution buffer composed of 50mM Hepes, pH 8.0 and 20mM reduced glutathione. Purity was assessed by SDS-PAGE and Comassie blue staining.

### Antibodies

Total Akt1/2/3 antibody was from Santa Cruz Biotechnology, HA antibody was from Sigma; anti-phospho-Akt (S129) and its not-phosphorylated counterpart were raised and purified as elsewhere described [22]. Secondary antibodies towards rabbit and mouse IgG, conjugated to horse radish peroxidase, were from PerkinElmer.

### Peptide synthesis

Peptide synthesis was performed using a multiple peptides synthesizer (SyroII, MultiSynTech GmbH). All peptide was synthesized using the fluoren-9-ylmethoxycarbonyl (Fmoc) strategy, at 0,05 mmol scale on Fmoc –Val Wang resin. For each coupling cycle, 250 mmol of Nα-Fmoc-amino acid, 500 mmol of N-Ethyldiisopropylamine (DIPEA) and 250 mmol equivalents of O-(7-azabenzotriazol-1-yl)-N,N,N′,N′-Tetramethyluronium hexafluorophosphate (HATU) [26] were used. Functional side-chains of bulding units were protected by tert-butyl (t-But), trityl (Trt) and 2, 2, 4, 6, 7-pentamethyldihydrobenzofuran-5-sulfonyl (Pbf) groups. Cleavage of the peptides was performed by reacting the peptidyl resins with a mixture containing trifluoroacetic acid/H2O/thioanisole/ethanedithiol/phenol (10 ml/0.5 ml/0.5 ml/0.25 ml/750 mg) for 2.5 h.

Crude peptides were purified by reverse phase HPLC on a preparative column (Prep Nova-Pak HR C18, Waters, USA). Molecular masses of the peptide were confirmed by mass spectroscopy on a MALDI TOF-TOF using an Applied Biosystems 4800 mass spectrometer.

### CK2 kinase assay

Phosphorylation of synthetic peptides was performed with recombinant CK2 α_2_β_2_ (0.025 μg) incubated at 30°C in a final volume of 20 μl containing 50 mM Tris/HCl (pH 7.5), 100 mM NaCl, 10 mM MgCl_2_, the indicated concentrations of peptides, and 20 μM [γ^33^P-ATP] (5000–10000 cpm/pmol). Assays were stopped by spotting onto phosphocellulose filters. Filters were washed four times in 0.5% orthophosphoric acid before counting.

Full length Akt phosphorylation experiments were performed on HA-Akt immunoprecipitated from overexpressing cells, incubated for 20 min at 30°C with 0.1 μg of recombinant CK2 α in the presence of a phosphorylation reaction mixture consisting of 25 mM Tris–HCl, pH 7.5, 10 mM MgCl2, 10 μM [γ-^33^P]ATP (~8000 cpm/pmol), in a total volume of 20 μl. Reactions were stopped by the addition of Laemmli loading buffer. Samples were boiled for 5 min, run on 11% SDS/PAGE and blotted on Immobilon-P membranes (Millipore); radioactive bands were detected and quantified by digital autoradiography (Cyclone plus storage phosphor system, PerkinElmer).

### PLK3 kinase assay

1.5 μg of purified recombinant-PLK3 were incubated with the substrate peptides for 10 min at 37°C in 20 μl of kinase assay buffer containing 20 mM Tris-HCl pH 7.5, 10 mM MgCl2, 5mM DTT (dithiothreitol), 20 μM [γ-^33^P]ATP (2000-6000 cpm/pmol). Assays were stopped by spotting onto phosphocellulose filters. Filters were washed four times in 0.5% orthophosphoric acid before scintillation counting.

### Phospho-aminoacid determination

For the identification of the amino acids phosphorylated by CK2 in Akt2 peptide, after phosphorylation samples were hydrolyzed by incubation for 4 h at 110°C in 6 N HCl, then subjected to high-voltage paper electrophoresis at pH 1.9 for 2.5 h [16], in the presence of nonradioactive phospho-amino acids, as migration reference. Radioactivity was detected by digital autoradiography (Cyclone plus storage phosphor system, PerkinElmer), while migration of standard references was detected by ninhydrin staining.

### Akt mutant production

We used expression vectors for the human HA-tagged Akt1 (pcDNA3.1- HA-Akt1) and HA-tagged Akt2 (pCMV6-HA-Akt2), as in [25]. HA-Akt1 S129A mutant plasmid was produced as previously described [22]; HA-Akt2 T132G mutant plasmids were obtained with the ‘QuickChange-Site Directed Mutagenesis’ Kit (Stratagene) using pCMV6-HA-Akt2 as template and two pairs of synthetic oligonucleotide primers each complementary to opposite strands of template:

5′-GGCTCCCCCAGTGACTCCTCCGGGACTGAGGAGATGGAAG-3′ and

5′-CTTCCATCTCCTCAGTCCCGGAGGAGTCACTGGGGGAGCC-3′.

The mutation was confirmed by sequencing analysis.

### Cell culture, transfection and lysis

HEK 293T cells were maintained in Dulbecco's modified Eagle's medium (DMEM; Sigma), supplemented with 10% Fetal Bovine Serum, 2 mM L-glutamine, 100 U/ml penicillin and 100 mg/ml streptomycin (all from Sigma), in an atmosphere containing 5% CO2.

The day before transfection, cells were plated into 6-well plates (2.5 × 10^5^ cells/well) and grown to about 70% confluence. Transient transfection was performed with 3 μg of plasmids for Akt1, Akt2, Akt1S129A and Akt2T132G by a standard calcium-phosphate procedure. The transfection mixture was removed after 16 h, and cells were collected 48 h after transfection. Cell lysis was performed as described in [22]. Protein concentration was determined by the Bradford method.

### Western blot and immunoprecipitation

Equal amounts of cellular proteins (10 μg) were loaded on 11% SDS/PAGE, blotted on Immobilon-P membranes (Millipore), processed by western blot with the indicated antibodies, and detected by chemiluminescence on a Kodak Image Station 440MM PRO. Quantitation of the signal was obtained by analysis with the Kodak 1D Image software.

Akt immunoprecipitations were performed from 50 μg of total protein lysates, from HA-tagged Akt isoforms transfected cells, in 30 μl of final volume. Lysates were incubated overnight at 4°C with 1.3 μl of anti-HA antibody followed by 25 μl protein A-Sepharose (Sigma-Aldrich) for 40 min at 4°C. Immunocomplexes were washed twice with Tris/HCl 50 mM (pH 7.5), once with Tris/HCl 5 mM (pH 7.5) and used as CK2 substrate in *in vitro* phosphorylation assays.

### Sequence analysis tools

The DNA sequences analysis were performed using the Basic Local Alignment Tool (BLAST) software of Uniprot. The blasted sequence used as a template consist of a fragment of 29 residues including the CK2 consensus phosphorylation site of human Akt1 (residues 110 to 146) and Akt2 (residues 117 to 145) respectively. A total of 42 of Akt1 and 43 of Akt2 consensus sequences were found and analyzed.

### In silico analysis

#### Protein-protein docking and molecular dynamics simulation

Protein-protein docking analysis was performed exploiting two FFT-based docking software PIPER and Zdock. During the procedures, Akt1/2 peptide models were considered as the probe and CK2 catalytic subunit (PDB code: 3Q04) as the target protein. 1000 complexes were obtained from both docking algorithms and clustered using the pairwise RMSD (Root Mean Square Deviation = 2.8 Å) into 6 largest clusters, to identify the most frequently sampled conformations. The final complex was chosen according to the solvatation energy scoring function. The selected complexes (parameterized with AMBER) were subjected to a Molecular Dynamics (MD) simulations using ACEMD (Acellera MC4-node system) [27]. The equilibration phase was performed by applying a 1 ns solvent (7710 explicit water molecules, TIP3P model; 150mM NaCl) equilibration with positional restraints on carbon atoms; Secondly 25 ns molecular dynamics was performed on the full system. The equilibration protocol used NPT ensemble (isothermal, isobaric), at constant pressure (1 atm, Berendsen method [28] and temperature (300 K, Langevin thermostat) [29]. Finally, a 300 ns molecular dynamics simulation (NPT, 1 atm, 300 K, as explained above) were performed to obtain the final interaction pattern of the complex.

#### Protein and peptides preparations

CK2 crystal structure (PDB code: 3Q04) was processed in order to remove ligands and water molecules. Hydrogen atoms were added to the protein structure using standard geometries with the MOE program [30]. To minimize contacts between hydrogens, the structures were subjected to Amber 99 forcefield minimization until the root mean square deviation of conjugate gradient was < 0.1 kcal mol^−1^ Å^−1^ keeping the heavy atoms fixed at their crystallographic positions.

To predict the structure of Akt1/2 peptides, a combination of different Neural Network based algorithms has been used (Jpred [31], PsiPred [32], Spider [33]). The final result was obtained through a consensus strategy where the prediction was confirmed at 90% by all the techniques considered, unless differently indicated. The peptides 3D structure were build in MOE [30] exploiting the secondary structure prediction and subjected to a short equilibration Molecular Dynamics protocol (ACEMD, 5ns of NPT, 1atm, 300K, explicit water) [27].

### Statistics

Statistical analysis was performed with the Software Graphad Prism. All data were collected from at least 3 independent experiments. Graphs show quantifications and SEM, image figures show representative experiments.

## Results

### 1. Phylogenetic analysis of sequences surrounding Akt1 S129 and Akt2 S131

We first wondered how conserved in different species the sequences surrounding Akt1 S129 and Akt2 S131 are (**Tables 1 and 2**).

Both S129 and the amino-acid sequence surrounding it – inclusive of the CK2 consensus S-x-x-E – are highly conserved across all Akt1 sequences (**Table 1**), consistent with the concept that its phosphorylation by CK2 must play a general function. The only few substitutions are mainly conservative (see E in -2 instead of D in some mammal species) and not expected to affect phosphorylation by CK2.

Akt2 S131 is also highly conserved as such, but the three aminoacids flanking it are different from their homologous around Akt1 S129, and the sequence downstream is rather variable across different species (see **Table 2**). To note, however, that the consensus for CK2 phosphorylation is conserved in nearly all Akt2s from different species, with just few exceptions. This is especially remarkable if the sequences surrounding S129 Akt1 and S131 Akt2 in human sequences are compared (see **Table 3**): in both, not only the minimum CK2 consensus (i.e. the E at +3) is present, but three additional acidic aminoacids are conserved (D at -2, and E at +4 and +6) which are often found at CK2 sites [1] and have been shown to empower site recognition by this kinase [11].

While it could be interesting to speculate why a site intended for being not phosphorylated by CK2 is nevertheless highly conserved as far as its CK2 consensus is concerned (see Discussion), the failure of CK2 to phosphorylate Akt2 S131 rises the question: which is/are the negative determinant(s) that prevent the phosphorylation of a site apparently optimally shaped for being targeted by CK2, in spite of the fact that a serine displaying an identical signature (D-X_1_**-S**-X_2_-X_3_-E-E-X_4_-E) is readily phosphorylated in Akt1?

### 2. Detrimental effect of T at n+1 on the phosphorylation of Akt2 by CK2

To provide a clear-cut answer to the above question, a set of peptides have been synthesized either exactly reproducing the amino acid sequence surrounding S129 and S131 in Akt1 and Akt2, respectively, or in which residues flanking the target serine, denoted above as X_1_, X_2_ and X_3_, which in Akt2 are S, T and T respectively, have been individually replaced by their homologous in Akt1 (N, G and A respectively). The complete list of the peptides synthetized and used in this work is shown in **Table 3**.

**Fig 1:**
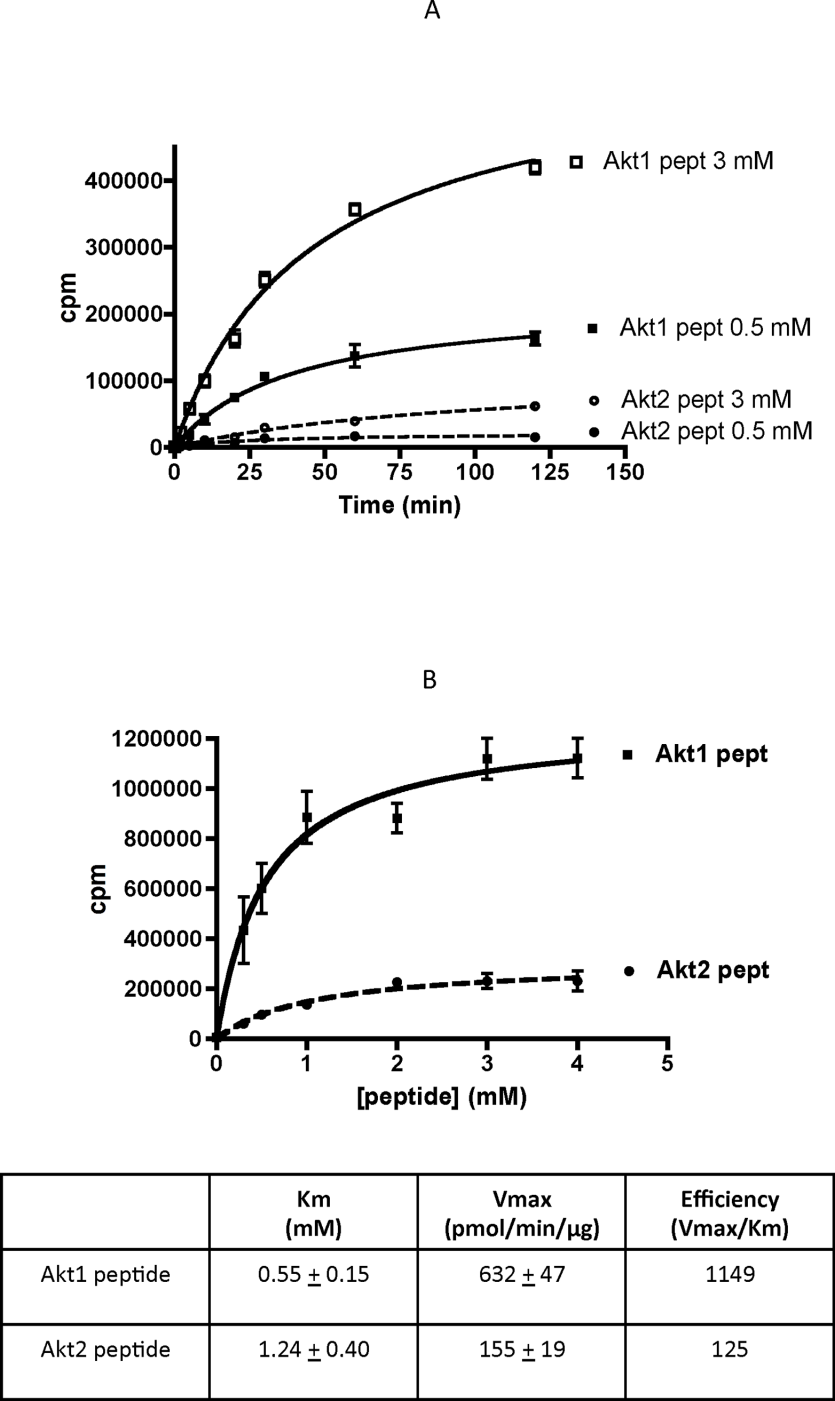
Phosphorylation of Akt1 and Akt2 peptides by CK2: Time course and kinetics. (A) Akt peptides at the indicated concentrations were incubated with CK2 for increasing length of times (0-2-5-10-20-30-60-90-120 min). (B) Akt peptides at increasing concentrations (0.3-0.5-1-2-3-4 mM) were incubated with CK2 for 10 min. Kinetics values were calculated by Prism Graphpad Software. Phosphorylation assay conditions as in Materials and Methods. Results are reported as means of 3 experiments ± SEM.

The time course phosphorylation experiment on display in **Fig 1A** shows that in contrast to the Akt1 wt peptide, which is a good substrate for CK2, the Akt2 wt peptide is only phosphorylated to negligible extent. This indicates that local negative determinant(s) are responsible for failure of Akt2 S131 to be phosphorylated by CK2, without having to assume higher level structural constraints. The superiority of the Akt1 peptide over the Akt2 peptide is also highlighted by the kinetics of **Fig 1B**, showing that the modest phosphorylation of the Akt2 peptide is accounted for by both higher Km and lower Vmax values, collectively responsible for an almost 10-fold drop in phosphorylation efficiency in comparison to the Akt1 peptide. Similar results were obtained with the monomeric CK2 α instead of the tetrameric α_2_β_2_ (not shown).

**Fig 2:**
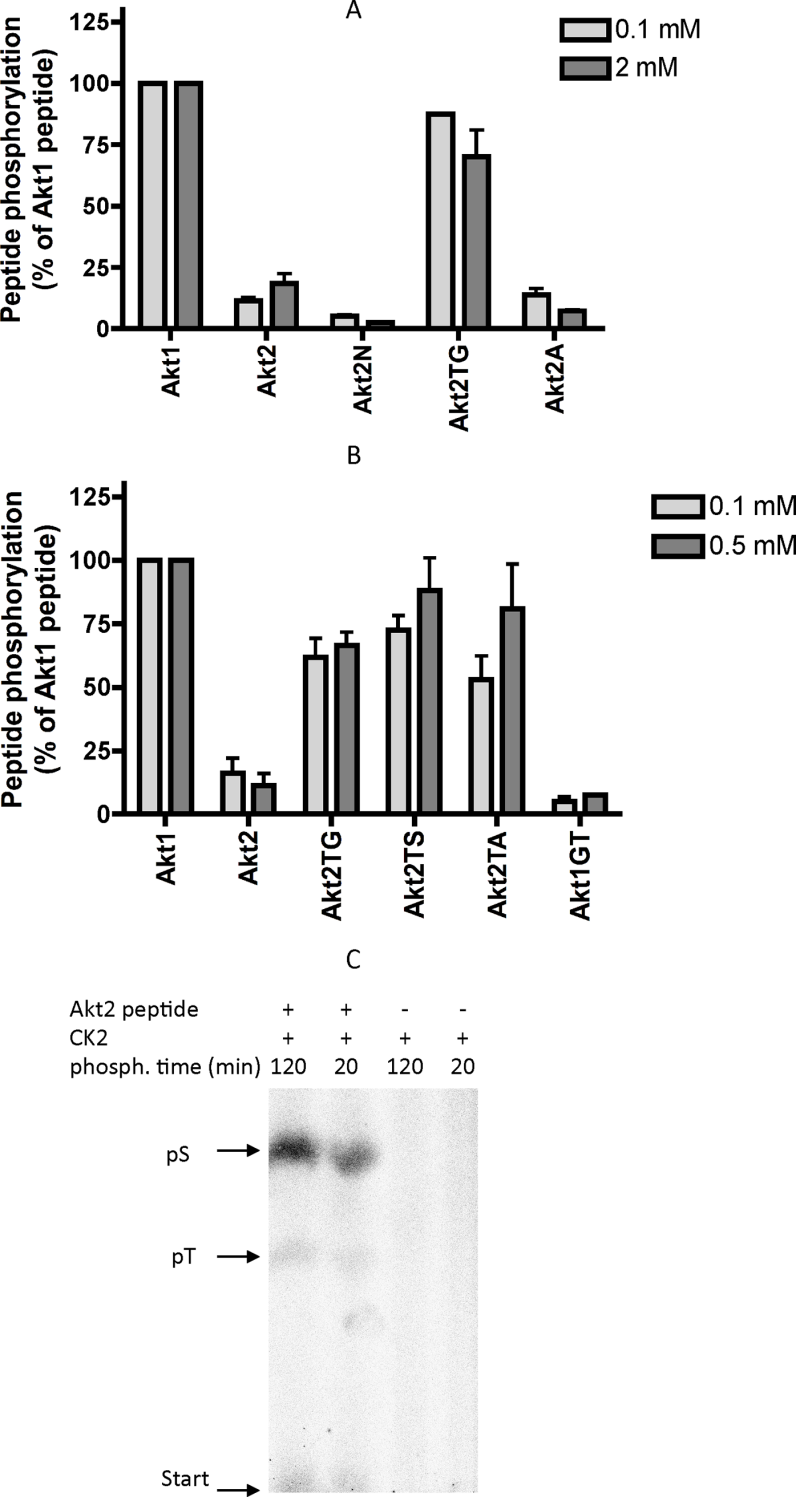
Phosphorylation of Akt1 and Akt2 peptide variants by CK2. (A) and (B): Peptides at the indicated concentrations were incubated with CK2 for 10 min, under conditions described in Materials and Methods. Results are reported as % of the cpm value obtained with Akt1 wt peptide (means of 4 experiments ± SEM). (C): CK2 was incubated with 2 mM Akt2 peptide for 20 or 120 min in a radioactive mixture; incubations were also performed in the absence of peptide, to evaluate possible contribution of CK2 autophosphorylation. Samples were hydrolyzed to obtain free phospho-aminoacids, that were separated by high voltage electrophoresis and detected by digital autoradiography. Migrations of standard phospho-aminoacids, as well as the starting point of the electrophoresis, are indicated.

As shown in **Fig 2A**, replacing in the Akt2 peptide S at n-1 with N (Akt2N peptide), and T at n+2 with A (Akt2A peptide) have no beneficial effect. By sharp contrast, the replacement of T at n+1 with G (Akt2TG peptide) confers a susceptibility to CK2 phosphorylation comparable to that of the Akt1 peptide.

The conclusion that indeed a threonine at position n+1 represents a specific local negative determinant responsible for failure of Akt2 S131 phosphorylation was corroborated by two additional observations: on one side also its substitution with other residues, namely S and A, is sufficient to substantially improve the phosphorylation of the Akt2 peptide (**Fig 2B**), on the other side the Akt1 peptide derivative in which G at n+1 has been replaced by T, is phosphorylated by CK2 as poorly as the Akt2 wt peptide (**Fig 2B**).

It should be also noted that although in some of the peptides listed in Table 3, and tested in Fig 1, notably the Akt2 wt peptide, a threonine residue is also present which fulfills the CK2 consensus (T-x-x-E), the modest phosphorylation is entirely accounted for by phospho-serine, with only faint traces of phospho-threonine detectable (**Fig 2C**).

**Fig 3:**
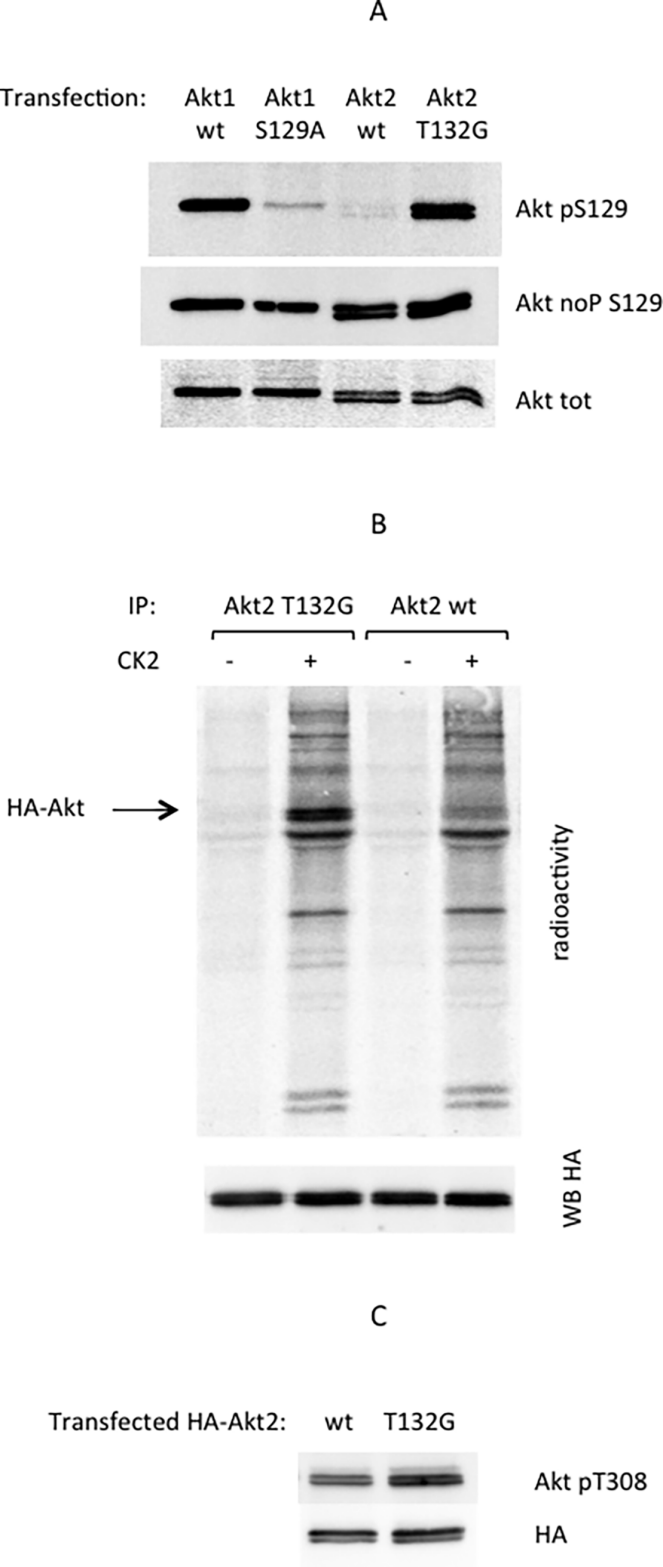
Effect of T132G mutation on the phosphorylation of full length Akt2 by CK2. (A): 10 μg proteins of total lysate from HEK 293T transfected cells were analyzed as indicated. Akt pS129 indicates development with phospho-S129 specific antibody (Akt1 S129A mutant was used as a negative control), Akt noP S129 indicates development with an antibody recognizing the same epitope but independently on phosphorylation, Akt tot indicates development with a generic Akt antibody, recognizing all Akt isoforms. (B): HA-Akt T132G or HA-Akt wt were immunoprecipitated with anti-HA from HEK 293T transfected cells. Immunocomplexes were incubated in a radioactive phosphorylation mixture, in the absence or in the presence of CK2. Samples were analyzed by SDS/PAGE and blotting, followed by digital autoradiography to detect radioactivity, and WB with HA antibody to ensure similar immunoprecipitation levels. (C): 10 μg proteins of total lysate from HEK 293T cells transfected for HA-Akt2 wt or HA-Akt2 T132G were analyzed by WB with Akt pT308 or HA antibody, as indicated.

The data presented so far provide the incontrovertible evidence that T132, adjacent to the C terminus of S131 in Akt2, is responsible for the very poor phosphorylation of the Akt2 peptide by CK2. To check if the same applies to the full length protein within its cellular environment, an Akt2 mutant has been generated in which T132 was replaced by G, i.e. the residue homologous to Akt2 T132 in Akt1, which is instead a good target of CK2. We expressed this mutant (Akt2-T132G) in HEK-293T cells (since these cells express significant amounts of CK2 and were already exploited to demonstrate the in vivo phosphorylation of Akt1 Ser129 [22]), and evaluated its phosphorylation at S131 by using an anti-Akt1(pS129) phospho-specific antibody. As shown in **Fig 3A**, at variance with wt Akt2 which was not recognized by this antibody, a detectable signal was evident in the lysate of cells expressing the Akt2 T132G mutant, revealing its endogenous phosphorylation.

Its in vitro phosphorylation by CK2 was also demonstrated by immunoprecipitation of Akt2 from cells expressing either the wt kinase or its T132G mutant, followed by incubation with CK2 and [γ^33^P]-ATP: as shown in **Fig 3B** only the latter underwent detectable phospho-radiolabeling by the kinase.

Since we have previously demonstrated that S129 phosphorylation promotes a higher Akt1 activity, at least in part due to a higher level of T308 phosphorylation [23], we investigated if this is also true for the T132G Akt2 mutant; the results, shown in **Fig 3C**, confirmed that the mutation of T132 to G, evoking the Akt2 phosphorylation at Ser131, is also accompanied by an increased phosphorylation of T308, compared to wt Akt2.

### 3. Effects of a T at n+1 position on the secondary structure

Once established that the sharply different susceptibility to CK2 phosphorylation of Akt2 and Akt1 is conferred by a threonyl residue adjacent to the target Serine in Akt2, the question we wanted to answer was why this feature has such a detrimental effect on Akt2 S131 phosphorylation, although all the acidic determinants for the site recognition by CK2 are still there.

**Fig 4:**
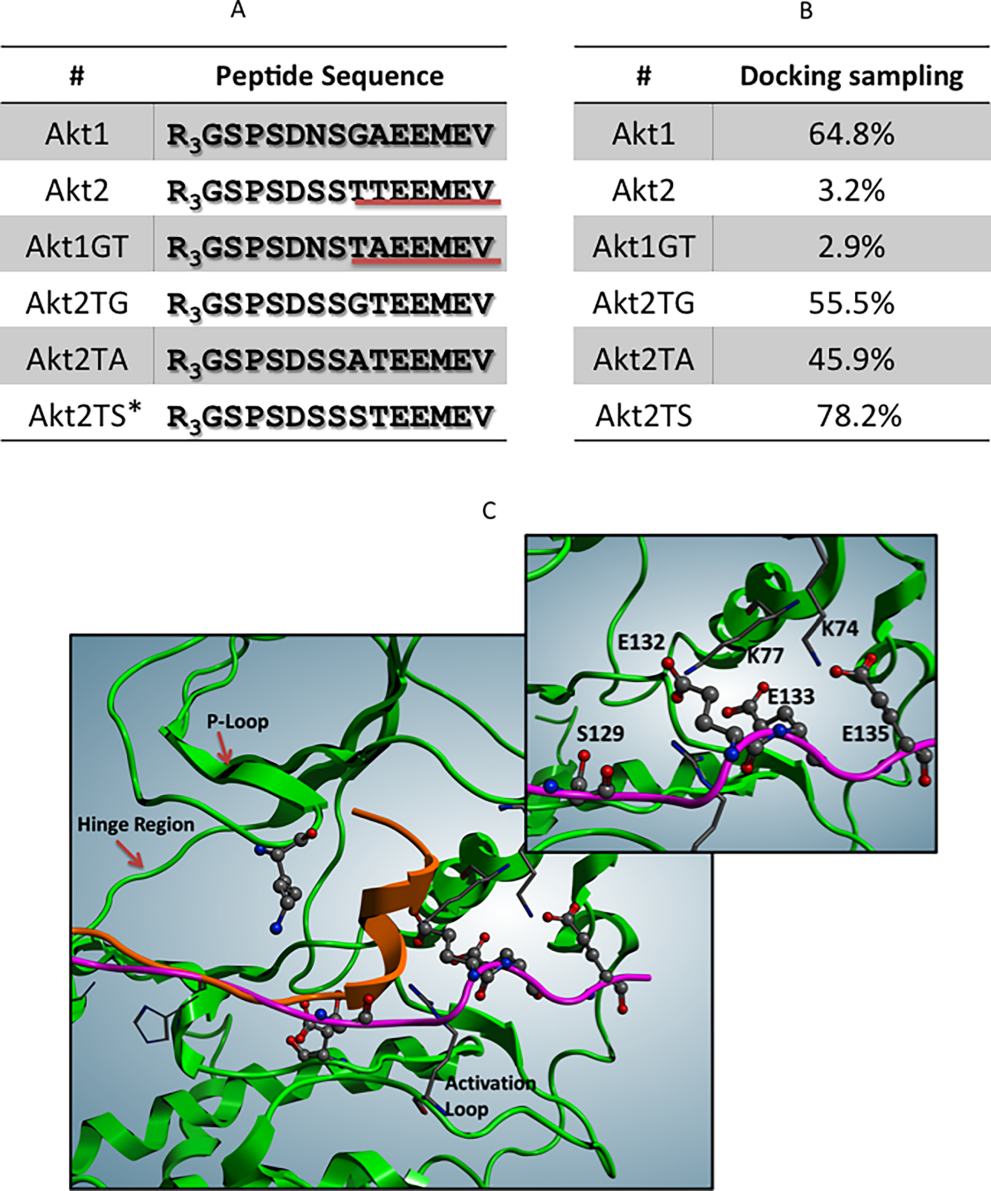
Effect of n+1 T on Akt2 secondary structure. (A) Predicted secondary structure for Akt1 / Akt2 peptide variants: α-helix is indicated by red underlining. The results from different algorithms (see Methods) were evaluated and considered acceptable only when consensus was at least 90%, with the exception of Akt2TS peptide, where (*) indicates that a consensus of 70% was considered. (B) Docking sampling of the most energetically favorable complexes clusterized by an RMSD < 3Å. (C) Protein-protein docking analysis and Molecular Dynamics simulation. Comparison between the Akt1 peptide (Violet) and Akt2 peptide (orange) docked against CK2α (green, PDB code: 3Q04); in the inset, the detailed interaction of Akt1 and CK2α substrate binding site is magnified.

To address this question, an in silico study was performed grounded on peptides secondary structure prediction, protein-protein docking analysis, and molecular dynamics simulation (**Figs 4)**. The structure prediction analysis of the peptides (**Fig 4A**) shows an α-helix organization for Akt2, while, on the contrary, the Akt1 peptide is entirely random coiled. This is consistent with the presence in +1 of a G, which displays low propensity to form α-helix, due to its high conformational flexibility. Indeed, the replacement of this G with T, as in the Akt1GT peptide, restores the α-helix organization, while the substitution of T in Akt2 with other residues (G, A, or S) prevents it.

When the Akt1, Akt2, and Akt1GT peptide models were docked against CK2α crystal structure (PDB code: 3Q04) through a protein-protein docking approach, we observed different percentage of docking sampling (**Fig 4B**): in the case of Akt1 peptide, 64.8% of the most energetically favorable complexes are compatible with a catalytic interaction between the target S129 and CK2 catalytic center (D156). On the contrary, the Akt2 peptide is docked close to the CK2 substrate binding site only in the 3.2% of the final sampling, and S131 is not correctly oriented against D156. Interestingly, the Akt1GT peptide displays a docking sampling percentage similar to Akt2 peptide (2.9%), confirming the major negative role of the T in +1. Consistently, the Akt2 peptide variants where T was replaced by G, A or S displayed docking sampling percentages similar to that observed for Akt1 peptide (55.5, 45.9 and 78.2%, respectively).

**Fig 4C** shows the superimposition of the Akt1 and Akt2 peptides docked against CK2α crystal structure (see Materials and Methods for details): in the case of Akt1, the peptide is lounged into CK2 substrate binding site with Akt1 S129 correctly oriented towards CK2 catalytic site (2.8 Å). Moreover, the glutamic residues downstream to the phosphorylatable S129 (E132, E133, E135) interact through electrostatic interactions with CK2α residues K74, K77, the well known lysines implicated in the recognition of CK2 substrates at downstream position [19]. On the contrary, S131 of the Akt2 peptide is not correctly oriented against D156; moreover, the presence of the α-helix downstream to S131 docks the peptide in an orientation that prevents the electrostatic interactions between its acidic residues and the key CK2 lysines K74/K77.

### 4. Are other protein kinases able to phosphorylate Akt2 S131?

**Fig 5:**
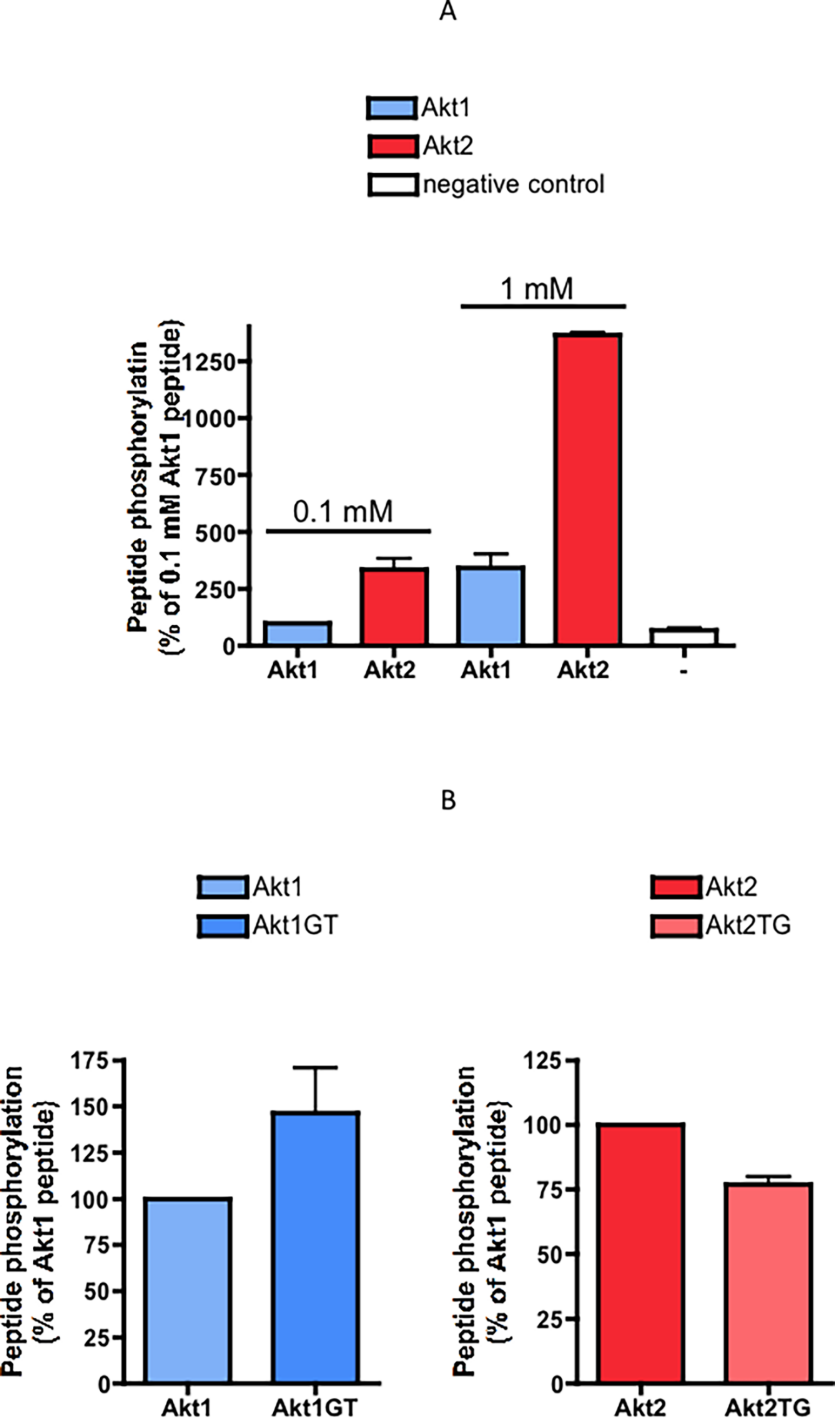
Phosphorylation of Akt1 and Akt2 peptides by PLK3. (A): Akt1 and Akt2 peptides at the indicated concentrations were incubated with PLK3, under conditions described in Materials and Methods. Results are reported as % of the cpm value obtained with Akt1 peptide at 0.1 mM (means of 3 experiments ± SEM). cpm detected with 1mM Akt2 peptide were in the range 50000-150000, according to the specific radioactivity; for comparison, a negative control, corresponding to activity in the absence of any peptide, is also shown in the graph (2800-9000 cpm). (B): wt and mutant Akt1 and Akt2 peptides were used at 0.6 and 1.2 mM as substrate in PLK3 assays. Results are reported as % of the cpm value obtained with each wt peptides at each concentration (means of 3 experiments ± SEM).

From the biochemical data and the bioinformatics analysis presented above, it appears that an individual residue, T132 replaced for its homologous residue in Akt1, G130, is responsible alone for local structural alterations that make Akt2 much less prone to CK2 phosphorylation as compared to Akt1. Intriguingly, however, this happens without any loss of the typical signature of CK2 sites conferred by a number of acidic residues which are conserved around the target serine in Akt2 as well as in Akt1, as also nicely illustrated by the observation that the phospho site specific antibody that recognizes Akt1 pS129 also recognizes Akt2 pS131 **(Fig 3A**). Lucubrating about this intriguing coincidence, we reasoned that survival of this phosphoacceptor site might reflect the intervention of another kinase, different from CK2, and, unlike CK2, committed to the phosphorylation of the linker region of both Akt1 and 2 under special circumstances. Pertinent to this may be two observations: firstly, a reduced yet still detectable phosphorylation of Akt1 S129 is occurring in C2C12 cells in which the whole CK2 catalytic activity has been knocked out by the CRISPR/Cas9 procedure [34], disclosing the existence of other kinase(s) able to phosphorylate this residue. Secondly, an aspartic acid is 100% conserved at position n-2 relative to the phosphorylatable Serine in both Akt1 and Akt2 (see **Tables 1 and 2**). An acidic residue in this position is welcome by CK2 but not required for its site recognition, while it may be of crucial relevance for the targeting by other kinases, especially those of the Polo-like kinase (PLK) subfamily [35]. Based on these observations, we performed in vitro assays on the Akt1 and Akt2 peptides with PLK3. Indeed, we observed an opposite ratio of phosphorylation: while the Akt1 peptide is by far preferred over the Akt2 one by CK2 (see **Figs 1A** and **1B**), the opposite is true of PLK3 (**Fig 5A**). Consistently, PLK3 does not perceive T in n+1 as a negative determinant, since its introduction instead of G in Akt1, or its replacement with G in Akt2, have no significant effect (**Fig 5B**).

This makes PLKs plausible candidates as phosphorylating agents of Akt2, although for the time being the phosphorylation of Akt2 at S131 remains a matter of conjecture.

## Discussion

The finding of a differential phosphorylation by CK2 of Akt1 and Akt2 [25] was hard to explain considering the conservation of the consensus sequence recognized by CK2 in both isoforms. Here we show that a single primary sequence element is responsible for such a diversity, disclosing for the first time that a T at n+1 position, as it occurs in the Akt2 sequence, is sufficient to compromise phosphorylation although the whole consensus sequence is conserved. A complete recovery of phosphorylation by CK2 is promoted by the replacement of this T with a G, as it is found in the Akt1 sequence, as well as by other residues, namely A, and S, despite this latter represents a conservative replacement with respect to T (**Fig 2**). The T to G mutation restores phosphorylation not only of the peptide but also of the full length Akt2, both *in vitro* and in cell (**Fig 3**).

The in silico analysis for structure peptides prediction and docking towards CK2α provides a rationale for this finding by showing that a T at n+1 (but not a G, as in Akt1) promotes an α-helix organization at the downstream side of the consensus, hampering the interaction between the crucial acidic side chain at position n+3 and the key residues of the CK2 active site committed to its binding (**Fig 4**).

The first practical relevance of our observation is a warning against cursory identification of CK2 sites, just based on the presence of an acidic determinant in +3. **Table 4**schematically summarizes whether the different peptides analyzed in this work are good substrates for CK2 (+) or not (-). In all of them the glutamic acid in +3 is preserved, while it is evident that the lack of significant phosphorylation correlates with the presence of a T in +1 position. This rises the question whether putative CK2 sites with the same motif (S-T-x-E/D) are bona fide targets of this kinase. In the Phosphosite.org page for CK2 substrates (http://www.phosphosite.org/), albeit uncommon, this motif is present in 12 site (out of the 760 total phospho-sites reported). It could be interesting to verify whether CK2 is their actual phosphorylating agent; in this case, the negative effect of a threonyl residue adjacent to the C-terminal side of the target serine, as observed in the case of Akt2 S131, has to be considered sequence-sensitive. Otherwise, we have to assume that kinases other than CK2, such as PLKs, are likely involved.

**Table 4:** Summary of the phosphorylation of Akt peptides by CK2. Peptides are listed as in Table 3, follow by (+) or (-) accordingly to their level of phosphorylation by CK2 (see text for details). The underlined residues are S129 of Akt1 and S131 of Akt2 of human sequence, respectively.

Peptide name	Sequence	Phosphorylation by CK2
**Akt1**wt	R_3_GSPSD**N**S**GA**EEMEV	+
**Akt2**wt	R_3_GSPSD**S**S**TT**EEMEV	-
**Akt2N**	R_3_GSPSD**N**S**TT**EEMEV	-
**Akt2A**	R_3_GSPSD**S**S**TA**EEMEV	-
**Akt2TG**	R_3_GSPSD**S**S**GT**EEMEV	+
**Akt2TA**	R_3_GSPSD**S**S**AT**EEMEV	+
**Akt2TS**	R_3_GSPSD**S**S**ST**EEMEV	+
**Akt1GT**	R_3_GSPSD**N**S**TA**EEMEV	-

In the second place, our findings definitely confirm that human Akt2 is not targeted by CK2. The present demonstration that S131 phosphorylation is prevented by a simple sequence element, makes it unlikely the occurrence of physiological conditions able to overcome this impasse. Akt1 and Akt2 are the two main ubiquitous isoforms of Akt; they share the same structural organization and mechanism of activation, and their functions are largely overlapped. However, they display also distinctive features and substrates, which are particularly relevant in explaining their isoform-specific roles [36–38]; their different susceptibility to regulation by CK2 can be particularly interesting in this context.

The absolute conservation of Akt1 S129 and surrounding sequence (**Table 1**) is consistent with a dominant role of this CK2 phosphorylation site in Akt1. We have already shown that S129 phosphorylation, besides upregulating active Akt1 [22], is instrumental for the recognition of palladin, an actin-associated protein involved in cancer cell migration and invasion [39,40], suggesting its implication in the determination of substrate selectivity. Perhaps other functions are still waiting to be disclosed.

Conversely, the Akt2 sequence around S131 is more variable (**Table 2**), suggesting that whenever present, its phosphorylation does not critically impinge on the selective pressure process. The CK2 consensus is in fact conserved, but in several organisms (underlined in **Table 2**), notably human, other primates, rodents, and elephant, its efficacy is abrogated by a T at position n+1. Rather, the possibility that kinases other than CK2 can phosphorylate this Akt2 site deserves attention, and major candidates are PLK kinases. Like CK2 also PLKs are acidophylic, but their consensus is mainly dictated by upstream determinants [35], and it is not disrupted by the T at n +1 as already shown by our results on display in **Fig 5**. Consistently the motif D-x-S, of crucial relevance for S131 recognition by PLKs, is conserved in Akt2s from all organisms. To the best of our knowledge, Akt2 S131 has never been found phosphorylated in literature or reported in phospho-sites database (for comparison, according to phosphosite.org, Akt1 S129 phosphorylation was found 40 times using only proteomic discovery – mode mass spectrometry, vs 0 times for Akt2 S131), and this suggests a sporadic occurrence of its phosphorylation. Nevertheless, it should be considered that, at variance with CK2, PLKs are not constitutively active: they play specific roles in mitotic entry and cell cycle, and they are controlled by complex mechanisms involving phosphorylation, proteolysis and transcription, depending on the biological context [41]. Thus, the possibility still remains that Akt2 S131 phosphorylation, at least in humans, is a finely tuned process, occurring only under specific and transient circumstances. Our present results pave the road toward future studies aimed at verifying its actual occurrence and significance.
